# Ant Plant (*Myrmecodia tuberosa*) Hypocotyl Extract Modulates TCD4+ and TCD8+ Cell Profile of Doxorubicin-Induced Immune-Suppressed Sprague Dawley Rats *In Vivo*

**DOI:** 10.3797/scipharm.1302-03

**Published:** 2013-06-21

**Authors:** Sumardi, Triana Hertiani, Ediati Sasmito

**Affiliations:** 1 Faculty of Pharmacy, Gadjah Mada University, Sekip Utara, 55281, Yogyakarta, Indonesia.; 2 Faculty of Pharmacy, Sumatera Utara University, Tri Dharma, 20155, Medan, Indonesia.

**Keywords:** Ant plant, *Myrmecodia tuberosa* hypocotyl, Doxorubicin, TCD4+, TCD8+

## Abstract

*Myrmecodia tuberosa* Jack (Rubiaceae) has been used as part of traditional Indonesian remedies for a wide range of therapeutic usages in West Papua. Our preliminary study revealed the significant potency of these plant extracts and fractions as an immunomodulator by an *in vitro* technique on Balb/c mice. This study explored the effect of *M. tuberosa* hypocotyl ethanol extract on the TCD4+ and TCD8+ cell profiles of doxorubicin (Dox)-induced immune-suppressed Sprague Dawley (SD) rats by an *in vivo* method. Dried powder of *M. tuberosa* hypocotyl was macerated in 95% ethanol. Following solvent evaporation in a vacuum, the ethanol extract (EE) was partitioned to yield an *n*-hexane fraction (FH) and residue (FNH). FNH was further partitioned to yield ethyl acetate (FEtOAc) and water fractions (FW). The extract and fractions in the concentrations 10, 20, 50, and 100 μg/mL were tested on macrophage cells by the latex bead method, while the proliferation of lymphocyte cells was evaluated by the MTT assay. The total phenolic and flavonoid contents of those fractions were evaluated. The active fraction was administrated orally on Dox-induced SD rats for 28 days by an *in vivo* method to observe the TCD4+ and TCD8+ cell profiles. The *in vivo* assay showed that the FNH could maintain the number of TCD4+ cells, but not the number of TCD8+ cells. The ED_50_ observed was 24.24 mg/kg BW. Steroid/terpenoid compounds were detected in this fraction along with the phenolics and flavonoids. The FNH contained 3.548 ± 0.058% GAE of total phenolics and 0.656 ± 0.026% QE of total flavonoids. *M. tuberosa* hypocotyl extract is a potent immunomodulatory agent and may act as co-chemotherapy in Dox use.

## Introduction

As part of their survival strategy, multicellular organisms developed a mechanism to protect themselves from infectious pathogens and at the same time, maintain the regulation of their own body cells. This defense mechanism is known as the body’s immune system and can be improved, maintained, or suppressed by agents called immunomodulators [1, 2].

TCD4+ lymphocyte cells or T helper (T_H_) and TCD8+ or T cytotoxic (Tc) cells are part of the adaptive immune system components that have important roles in eradicating infection and abnormal cells [1]. TCD4+ cells help B cells to produce antibodies, enhance and maintain TCD8+ cell responses, regulate macrophage function, control the immune response against various types of pathogenic microorganisms, and enhance or suppress immune responses to control autoimmunity and abnormal cells [3]. Moreover, TCD4+ cells can reject TCD8+ cells that are resistant to tumor cells (Diez *et al*., 2007). TCD4+ cells are required for the formation of TCD8+ cell memory, which includes division, cytokine secretion, self-renewing, and secondary immune responses [4].

Doxorubicin (Dox) is a drug of choice in many cancer therapies. Unfortunately, its damaging effects not only occur on cancer cells, but also to normal ones [5]. Dox can affect the immune system by decreasing interleukin-2 (IL-2) and production of interferon-γ (INF-γ), natural killer cells, lymphocyte cells, the CD4+/CD8+ ratio, as well as damaging the thymus organ [6, 7]. The search for agents to minimize the side effects is urgently required. Natural resources are good candidates for new drug sources. Zhang *et al*. (2005) for example, had reported that grape seed could improve immune system function in the presence of Dox [6].

*Myrmecodia tuberosa* Jack (Rubiaceae) is an indigenous plant used by the people of West Papua (Indonesia) to cure various diseases including cancer [8]. *M. tuberosa* is one of three ant plants which are widely used as part of natural healing remedies in West Papua [9]. Two species of insects i.e., the formica and myrmica species have been identified as symbionts of the sample plant. Those insects play a role as protector against the threat of predators and fungal infections [10, 11]. Ants have been reported to produce the immune-active compounds as part of a self defense mechanism [12]. Those active compounds may therefore contribute to the ant plant’s biological activity.

The extract and fractions of the hypocotyl of *M. tuberosa* could increase phagocytosis of macrophages and proliferation of lymphocyte cells on Balb/c mice by an *in vitro* method. The active fraction showed a presence in phenolics, flavonoids, steroid/triterpenoid, and conjugated double bond compounds [13]. High polyphenol content was expected to overcome Dox’s immunosuppresive effect. Flavonoids and phenolics have been reported to modulate inflammation-related macrophages (reviewed in [14]). Further analyses of this plant’s potency as an immunomodulator, specifically as complementary to Dox treatment, should be done by *in vivo* assays.

For determining the active fraction and dose to be tested *in vivo*, the effects of the crude extracts and fractions of *M. tuberosa* hypocotyls were evaluated by an *in vitro* method on SD rats. At the same time, the effects of total phenolics and flavonoids in each fraction were evaluated to determine the correlation to the activity. Afterwards, the active fraction was subjected to SD rats to examine the effect on the TCD4+ and TCD8+ cell profile by using an *in vivo* technique. Those rats were conditioned with Dox to induce an immune-suppressing effect.

## Results and Discussion

The extract and fractions of *M. tuberosa* showed the ability to increase the phagocytic activity of macrophage cells (Table 1). The ethanol extract (EE) at a dose of 20 μg/mL exhibited the highest activity and showed no significant difference from the positive control (LPS) at 10 μg/mL. Nevertheless, no dose-dependent relationship was observed.

The FEtOAc had the highest ability to enhance the proliferation of lymphocyte cells in comparison to the other fractions (Table 2). Also, an increase in the stimulation index (IS) trend was observed which was in line with increasing dose. The *n*-hexane fraction (FH) showed relatively lower activity compared to the other fractions, but significantly higher than the normal control.

The extract and fractions of *M. tuberosa* increased the phagocytic activity of the macrophage cells, but without a dose-dependent relationship. On the other hand, the extract and fractions could enhance lymphocyte proliferation, as it showed increased SI values with increasing dose. These results were in accordance with our previous report in Balb/c mice [13].

The gallic acid calibration curve was determined as y = 0.002× + 0043, R^2^ = 0.991. The EE showed the highest total phenolic content (3.75%). The FNH and the FetOAc fractions showed total phenolic content of 3.54% and 0.73% Gallic Acid Equivalent (GAE), respectively. The FH had an undetectable phenolic content (Table 3). The quercetin calibration curve was determined as y = 0.001× + 0008, R^2^ = 0.999. FEtOAc showed the highest total flavonoid content (2.15%), followed by EE, FNH, FW, and FH i.e., 1.11, 0.65, 0.094, and 0.088% Quercetin Equivalent (QE), respectively.

**Fig. 1:**
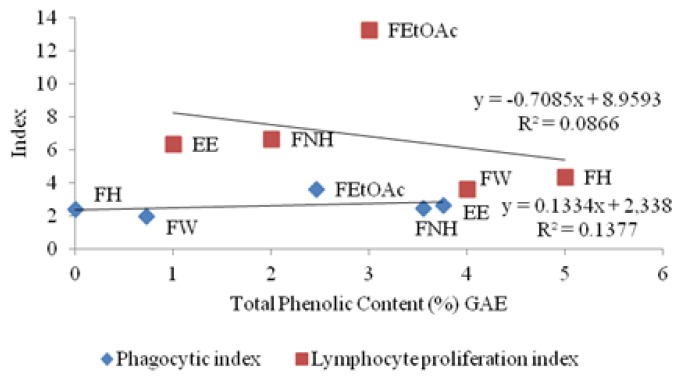
Correlation chart of total phenolic content (%) vs Phagocytic Index and Lymphocyte proliferation index

**Fig. 2:**
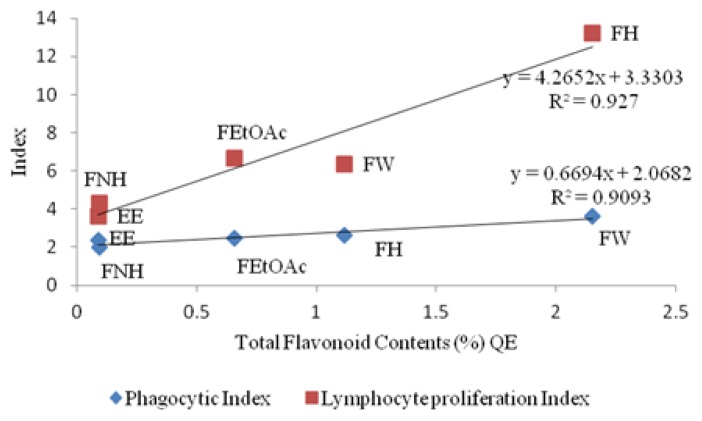
Correlation chart of total flavonoid content (%) vs Phagocytic Index and Lymphocyte proliferation Index

Correlation studies of total phenolic contents vs immunomodulatory effects (Fig. 1) revealed a weak correlation of both lymphocyte proliferation and macrophage phago-cytosis effects. On the other hand, the total flavonoid contents had a strong positive correlation with the immunomodulatory effects (confidence limit 95%, Fig. 2). Never-theless, the *n*-hexane fraction (FH) showed the least immunomodulatory effects. The fact that the FH was not active and possessed the least total phenolic and flavonoid content in comparison to other fractions, supports the aforementioned suggestion that the phenolics and flavonoids played major roles in the immunomodulatory activity of *M. tuberosa* hypocotyl.

The above result directed the FNH to be further explored in *in vivo* assays. Phytochemical analyses of the FNH performed on precoated silica gel F254 with ethanol:toluene (1:3) v/v as the mobile phase revealed the presence of steroid/terpenoids, phenolics, and flavonoids as chemical constituents (Table 4).

The amounts of TCD4+ and TCD8+ cells were measured by flowcytometer on day 14 and 28 (Tables 5–6). Statistical analyses of TCD4+ cell amounts measured on day 14 of the experiment showed no significant differences among the test groups in the baseline and negative control groups (Table 7). The TCD4+ precentage of the test group having 50 mg/kg BW measured on day 28 was increased in comparison to the negative controls. This dose might act as the optimum dose as shown by decreased effects on TCD4+ numbers observed in the test groups having 100 and 20 mg/kgBW doses. The ethno-medicine application of this herb is by decocting 10 g of the dried herb in approx. 250 mL water and drinking it as tea, two or three times a day. Some herbal products recommend the usage of the extract as 900 mg twice a day. A dose of 100 mg/kg of rat having 200 g BW can be converted to humans (70 kg) being a 1.1 g application of FNH daily.

The number of TCD8+ was decreased at the fourth week of the experiment in all groups. The baseline group showed a slight increase in the TCD8+ number measured on day 28 in comparison to day 14. This suggests that the FNH has lost its ability to increase or maintain the number of TCD8+ after the fourth week of treatment. Nevertheless, the TCD4+/TCD8+ ratio of the FNH test groups were higher than the baseline and negative control on day 28. The number of TCD4+ cells increased significantly after treatment with the FNH and showed an ED_50_ at 24.24 mg/kg body weight.

The content of flavonoids was considered to play a major role in the activity. Flavonoid glycosides increased the number of TCD4+ cells in the immune-suppressed mice [15]. However, the inability of the FNH to maintain the number of TCD8+ cells might be due a coexistence of other constituents, i.e. steroids/terpenoids, which may act as immune-suppressive agents. As reported by Fan et al. (2009), a triterpenoid compound, pachymic acid did not affect number TCD4+ cells, but decreased the number TCD8+ cells [16]. Further experiments are required to prove this suggestion. Nevertheless, the analyses of the TCD4+/TCD8+ ratio showed that the test groups caused significant improvement on day 28 in comparison to the baseline and negative control groups (Table 7). Considering that a decrease in the TCD4+/TCD8+ ratio is one of the known side effects of doxorubicin treatment (Zhang et al., 2005), the ability of FNH to increase this ratio supports its usage as a complementary agent in doxorubicin chemotherapy [6].

## Experimental

### Sample Preparation

*M. tuberosa* hypocotyls (3–4 months) were collected from Babo, Bintuni, West Papua (Indonesia) in February-March 2010. Taxonomy identification was done in the Dept. of Pharmaceutical Biology, Faculty of Pharmacy, University of Gadjah Mada, Indonesia as stated in the Plant Identification Letter Nr. BF/14/Ident/Det/I/2012, Reg. Nr. 733 and the voucher specimen was kept in the Green House of Pharmaceutical Biology Department, Faculty of Pharmacy, UGM, Indonesia. Plant determination was conducted according to Backer and Van Den Brink [17].

Hypocotyls were sorted and washed with water, sliced (3 mm thickness), oven-dried (Memmert, German) at 40–60°C, and milled. Dried powders were macerated in ethanol 95%, followed by solvent evaporation. Crude extract (EE) was then fractionated by liquid-liquid partitioning with *n*-hexane 90% methanol to yield the *n*-hexane fraction (FH) and the residue (FNH). After solvent evaporation, FNH was further partitioned by ethyl acetate-water to yield the acetate fraction (FEtOAc) and water fraction (FW).

Each extract and fraction was diluted with 0.5% Tween 80 to yield concentrations of 10, 20, 50, and 100 μg/mL. Dosages of 20, 50, 100 mg/kg BW in 0.5% Tween 80 were used for the *in vivo* assay.

### Macrophage Isolation and Phagocytic Activity Assay

Macrophages were isolated from SD rat peritoneal fluid by adding 10 mL of cold RPMI 1640. The aliquot was centrifuged at 1200 rpm 4°C for 10 min. About 3 mL of RPMI 1640 complete media (contains FBS 10% (v/v)) was added to the sediment clumps. The cells were counted by the Neubauer haemocytometer and then resuspended in complete media to obtain a cell suspension with 1.38×10^6^ mL^−1^ density. The cell suspension was then inoculated on 24-well microtiter plates (Nunc) which were covered by round cover slips. Each well contained a 200 μL suspension (2.8×10^5^ cells). The cells were incubated in a 5% CO_2_ incubator at 37°C for 30 min, and 1 mL of complete medium was added into each well and reincubated for another 2 h. Afterwards, the cells were washed twice with RPMI 1640 and then 1 mL of complete media was added, followed by 24 h incubation. Non-specific phagocytic activity measurements were performed by using 3 μm latex beads resuspended in PBS to get a concentration of 2.5 × 10^6^ mL^−1^. The 24 h-cultured peritoneal macrophages were washed twice with RPMI 1640. The latex suspension (200 μL/wells) and samples (200 μL/wells) were added. The suspensions were then incubated in 5% CO_2_ incubator at 37°C for 60 min. The cells were washed three times with PBS to eliminate excess latex beads. After drying at room temperature, fixation was done with methanol for 30 sec. Afterwards, the methanol was aspired and cover slips were left to dry, followed by 2% Giemsa (v/v) staining for 20 min. After being washed with distilled water, the cover slips were left to dry.

The amount of macrophages which phagocyted the latex beads, as well as the amount of latex beads consumed by the macrophages, were counted under an inverted microscope to calculate the macrophage phagocytic index. The positive control used was 10 μg/μL LPS [18, 19].

### Lymphocytes Isolation and Proliferation Assay

Spleen tissue was isolated aseptically from the SD rats and subsequently transferred to a 50 mm petri dish containing 10 mL of RPMI 1640 to yield the lymphocyte suspension in medium. After centrifugation at 3200 rpm, 4°C for 4 min, the clumps were separated from the supernatant and resuspended in 5 mL tris ammonium chloride buffer and left at room temperature for 15 min. RPMI was added to reach 10 mL suspension and centrifugation was repeated for another 4 min. The clumps were washed twice with RPMI and then diluted with complete media. The lymphocyte cell number was counted by a haemocytometer. Cells were kept in a 5% CO_2_ incubator at 37°C. Suspensions of the lymphocyte cells in 100 μL media (1.5×10^6^ mL^−1^) were distributed into 96-well microtiter plates (Nunc). Afterwards, 10 μL of hepatitis B vaccine (Havrix^®^, GlaxoSmithKline) was added into each well and incubated in 5% CO_2_ at 37°C for 24 h. After the sample suspensions were added, incubation was continued for another 48 h. Following 10 μL addition of 5 mg mL^−1^ MTT [3-(4,5-dimethylthiazol-2-yl)-2,5-diphenyltetrazolium-bromide] into each well, incubation took place at 37°C fo 4 h. Viable cells reacted with MTT to form a purple color. Reagent stopper (10% SDS) in 50 μL of HCl 0.01 N was added into each well.

To calculate the stimulation index of proliferation, absorbances were measured by using a microplate reader (Bio-Rad Benchmark, Japan) at 550 nm. The positive control used was 10μg/μL PHA (phytohaemagglutinin, Merck, Germany) [20, 21].

$${\text{SI}}_{\text{proliferation}}={\text{OD}}_{\text{sample}}/{\text{OD}}_{\text{normal}}$$

### Phytochemical Analyses

Several TLC systems were evaluated to get the best separation of the active fraction’s chemical contents. UV 254 and 366 nm lamps were used to detect the compounds with chromophores. Spray reagents i.e., vanillin-sulfuric acid, Cerium(IV)-sulfate, Liebermann-Burchard, Dragendorff, citroboric acid, and ammonia vapor were used to detect the chemical contents based on functional groups [22].

### Determination of Total Phenolic Content

Total phenolic content of the extract and fractions was determined by the Folin-Ciocalteu method [23]. With 0.1 mL of the sample and standard solutions, each was oxidized with 0.1 mL of the Folin-Ciocalteu reagent. After 4 min, the solutions were neutralized by the addition of 1 mL of 7.5% Na_2_CO_3_. After 120 min, 3.8 mL distilled water was added. Absorbances were measured at 751 nm. Total phenolic contents were calculated as gallic acid and done in triplicate.

### Determination of Total Flavonoid Content

Total flavonoid content of the extract and fractions was determined by the aluminum chloride colorimetric method [24]. Into 0.2 mL of each sample and standard solution were added 3.6 mL of ethanol 95%, 0.1 mL AlCl_3_ 10%, 0.1 mL potassium acetate 1 M, and 1 mL distilled water. After shaking the solution to obtain homogeneity, it was incubated at room temperature for 30 min. Afterwards, absorbencies were determined at 431 nm. Total flavonoid contents were calculated as quercetin and done in triplicate.

### Determination of TCD4+ and TCD8+ Cells Profile of Dox-Induced Immune-Suppressed SD Rats in vivo

Two-month-old Spraque Dawley (SD) male rats having a body weight of 150–200 g were placed in separated cages according to the groups. The cages were maintained at 28–32 °C and relative humidity of 98%. BR2 pellets were given to feed the animals and water was given *ad libitum*. The animals adapted to the experimental cages for one week before the treatment. Body weights were measured weekly. The animals were sacificed at the end of experiment to evaluate the toxicological effects (reported separately).

SD rats were divided into five groups, each consisting of five individuals. One group was selected as a baseline, three groups as test groups, with each receiving sample dosages of 20, 50, 100 mg/kg (p.o), and the one group treated with 10 mg/kg BW (i.p) Dox was used as a negative control. Test samples were given every day for 28 days, while Dox was given on days 1, 14, and 28. Blood samples were taken at days 14 and 28 through the eye orbital vein.

Determination of the TCD4+ and TCD8+ lymphocyte cells profiles were done by using the Flow Cytometry with Tricount methods. Measurements were performed in the laboratory of Clinical Pathology, Faculty of Medicine, Universitas Gadjah Mada, Indonesia according to the laboratory procedure [25].

### Statistical Analyses

The data obtained were analyzed by ANOVA (software SPSS 14.0) with *post hoc* Duncan’s test.

Results of the total phenolic content as well as total flavonoid content were analysed for the correlation to the immunomodulatory effects by the Pearson correlation coefficient with a significance level at 0.05.

## Figures and Tables

**Tab. 1:** Effect of extract and fractions of *M. tuberosa* on phagocytic activity of macrophage cells. Phagocytic activity was determined as Phagocytic Index (IP), Normal Control = 0.26 ± 0.06; n = 3

Treatment	IP (Mean ± SD)			

	10 μg/mL	20 μg/mL	50 μg/mL	100 μg/mL
EE	4.84 ± 0.18	2.24 ± 0.02	5.61 ± 0.39	2.64 ± 0.07
FNH	2.32 ± 0.27	5.73 ± 0.30	2.70 ± 0.24	2.46 ± 0.13
FetOAc	4.64 ± 0.78	2.76 ± 0.28	3.30 ± 0.35	3.61 ± 0.39
FH	2.30 ± 0.03	4.17 ± 0.59	4.73 ± 0.11	2.39 ± 0.14
FW	3.78 ± 0.15	3.06 ± 0.24	2.29 ± 0.08	1.99 ± 0.13

EE: Ethanolic extract; FNH: Nonhexane fraction; FEtOAc: Ethyl acetate fraction; FH: Hexane fraction; FW: Water Fraction.

**Tab. 2:** Effect of extract and fractions of *M. tuberosa* on proliferation of lymphocyte cells. Proliferation of lymphocyte cells was determined as the Stimulation Index (IS), Control Normal = 1.0 ± 0.013; n = 3

Treatment	IS (Mean ± SD)			

	10 μg/mL	20 μg/mL	50 μg/mL	100 μg/mL
EE	5.508 ± 0.017	5.136 ± 0.023	4.593 ± 0.020	6.339 ± 0.040
FNH	6.017 ± 0.038	2.898 ± 0.004	5.085 ± 0.019	6.661 ± 0.029
FetOAc	5.678 ± 0.050	4.424 ± 0.005	8.610 ± 0.015	13.237 ± 0.035
FH	5.678 ± 0.080	3.322 ± 0.015	4.000 ± 0.006	3.610 ± 0.020
FW	3.085 ± 0.029	4.424 ± 0.005	3.356 ± 0.013	4.322 ± 0.015

EE: Ethanolic extract; FNH: Non hexane fraction; FEtOAc: Ethyl acetate fraction; FH: Hexane fraction; FW: Water Fraction.

**Tab. 3:** Total phenolics and flavonoid contents of extract and fractions of *M. tuberosa.*

Sample	Total Phenolics Content (% GAE)	Total Flavonoids Content (% QE)
EE	3.757 ± 0.024	1.116 ± 0.255
FNH	3.548 ± 0.058	0.656 ± 0.026
FetOAc	2.458 ± 0.034	2.153 ± 0.135
FH	0.000 ± 0.012	0.088 ± 0.022
FW	0.729 ± 0.032	0.094 ± 0.021

Total phenolic content was calculated as gallic acid, total flavonoid content was calculated as quercetin, means ± SD, n = 3; Ethanolic extract; FNH: Non hexane fraction; FEtOAc: Ethyl acetate fraction; FH: Hexane fraction; FW: Water Fraction; GAE: gallic acid equivalent; QE: quercetin equivalent.

**Tab. 4:** TLC profiles of the FNH of *M. tuberosa* (mobile phase: ethanol : toluene (1 : 3 v/v) and stationary phase: aluminium precoated TLC silica gel 60 F 254)

Detection Method	hRf value							
UV 254 nm	40 (br)	50 (br)	–	–	–	–		
UV 366 nm	31 (yf)	38 (yf)	41 (yf)	44 (pu)	50 (yf)	54 (yf)	90 (yf)	96 (yf)
Cerium/H_2_SO_4_	21 (br)	39 (br)	41 (br)	46 (br)	50 (br)	52 br)	55 (br)	58 (br)
Vanillin/H_2_SO_4_	14 (bl)	25 (br)	29 (br)	40 (pu)	45 (br)	–	–	–
Liebermann- Burchard	17 (bl)	36 (br)	47 (pu)	54 (br)	–	–	–	–
Citroboric acid	47 (bl)	56 (br)	57 (br)	–	–	–	–	–
Ammonia	47 (y)	–	–	–	–	–	–	–
AlCl_3_	58 (y)						–	–
Dragendorff	–	–	–	–	–	–	–	–
FeCl_3_	57 (br)	–	–	–	–	–	–	–

br = brown; bl = blue; pu = purple; y = yellow; yf = yellow fluorescent.

**Tab. 5:** Amounts of TCD4+ and TCD8+ cells measured by Flowcytometry on day 14

Sample	N	TCD4+ of Lymph (%)	TCD8+ of Lymph (%)
Base Line	1	49.68	25.98
	2	54.06	34.96
	3	51.53	8.02
	4	53.95	9.79
	5	60.76	10.77

Negative Control	1	69.05	5.65
	2	n.t.	n.t.
	3	63.02	16.05
	4	66.10	18.55
	5	66.93	12.51

FNH 20 mg/kg body weight	1	54.09	30.23
	2	68.30	24.19
	3	68.44	23.90
	4	68.25	13.39
	5	64.26	23.87

FNH 50 mg/kg body weight	1	62.55	24.45
	2	56.63	15.48
	3	66.48	31.55
	4	64.43	32.61
	5	72.73	26.1

FNH 100 mg/kg body weight	1	61.27	24.22
	2	50.74	26.82
	3	73.82	20.53
	4	60.80	28.80
	5	63.15	26.85

n.t.: not tested, animals died.

**Tab. 6:** Amounts of TCD4+ and TCD8+ cells measured by Flow cytometry on day 28

Sample	Code	TCD4+ of Lymph (%)	TCD8+ of Lymph (%)
Base Line	1	27.44	18.15
	2	32.27	21.98
	3	44.81	19.54
	4	45.93	25.05
	5	47.55	20.05

Negative Control	1	n.t.	n.t.
	2	n.t.	n.t.
	3	22.4	5.45
	4	37.44	10.65
	5	9.62	12.19

FNH 20 mg/kg body weight	1	66.12	15.92
	2	49.13	9.24
	3	56.82	13.57
	4	n.t.	n.t.
	5	n.t.	n.t.

FNH 50 mg/kg body weight	1	71.67	10.01
	2	56.73	15.69
	3	87.25	11.31
	4	n.t.	n.t.
	5	n.t.	n.t.

FNH 100 mg/kg body weight	1	60.20	11.45
	2	39.96	11.82
	3	57.4	10.77
	4	72.59	14.51
	5	n.t.	n.t.

n.t.: not tested, animals died.

**Tab. 7:** TCD4+ and TCD8+ cells measured on day 14 & day 28 (means ± SD, α = 0.2)

Sample	TCD4+ of lymph (%)		TCD8+ of lymph (%)		TCD4+/TCD8+	
	Day 14	Day 28	Day 14	Day 28	Day 14	Day 28
Base line	54.00 ± 4.20	39.60 ± 9.11	17.90 ± 11.94	20.95 ± 2.67	1.36	1.89
Negative Control	66.28 ± 2.50	23.15 ± 13.92	13.19 ± 5.60	9.43 ± 3.53	5.03	2.45
FNH 20	64.67 ± 6.17	57.35 ± 8.51	23.12 ± 6.07	12.91 ± 3.39	2.80	4.44
FNH 50	64.56 ± 5.86	71.88 ± 15.26	26.04 ± 6.85	12.34 ± 2.98	2.48	5.82
FNH 100	61.96 ± 8.21	57.53 ± 13.45	25.44 ± 3.19	12.14 ± 1.64	2.44	4.74
